# Effects of repetitive transcranial magnetic stimulation on quality of postoperative recovery following major abdominal surgery: a study protocol for a single-center, prospective, randomized controlled trial

**DOI:** 10.3389/fmed.2026.1923206

**Published:** 2026-10-21

**Authors:** Junwen Tu, Shirong Wei, Yubin Shi, Sitong Zhou, Tong Zhi, Ming Yao, Huadong Ni

**Affiliations:** 1 Department of Anesthesia Medicine, Zhejiang Chinese Medical University, Hangzhou, Zhejiang, China; 2 Department of Anesthesiology and Pain Research Center, The Affiliated Hospital of Jiaxing University, Jiaxing, China

**Keywords:** dorsolateral prefrontal cortex, major abdominal surgery, quality of recovery-15 scale, randomized controlled trial, repetitive transcranial magnetic stimulation

## Abstract

**Background:**

Major abdominal surgery is a primary therapeutic strategy for various abdominal diseases, but it is often related to severe postoperative pain, complications, and delayed recovery. Repetitive transcranial magnetic stimulation (rTMS), a noninvasive neuromodulatory technique, may influence pain processing, emotional regulation, and other neural functions relevant to postoperative recovery. However, its role in improving quality of postoperative recovery remains insufficiently investigated, particularly with respect to patient-reported recovery outcomes.

**Methods/design:**

This investigation is a single-center, prospective, randomized controlled study. A total of 208 participants undergoing major abdominal surgery will be classified into receiving either active or sham rTMS in a random manner. Active stimulation will be delivered to the dorsolateral prefrontal cortex (DLPFC) at 120% of the resting motor threshold (RMT) and 10 Hz. The primary outcome is the Quality of Recovery-15 (QoR-15) score at 24 h postoperatively. Exploratory secondary outcomes include QoR-15 scores at additional time points, pain intensity, delirium incidence, opioid consumption, postoperative nausea and vomiting (PONV), anxiety, patient satisfaction, and gastrointestinal recovery. Safety outcomes include adverse events (AEs). We will conduct the primary analysis according to the intention-to-treat (ITT) principle, comprising all randomized participants.

**Discussion:**

This investigation is designed to estimate whether rTMS can enhance quality of postoperative recovery in participants experiencing a major abdominal operation. The outcomes will provide evidence about the potential role of rTMS as a noninvasive adjunct in perioperative care and inform future multicenter trials.

**Clinical Trial registration:** https://www.chictr.org.cn/index.html, identifier ChiCTR2600119250.

## 1 Introduction

Amid the growing global cancer burden, major abdominal surgery remains the primary treatment for gastrointestinal malignancies, including gastric and colorectal cancers, and critically influences survival and postoperative quality of life (1). Minimally invasive surgical procedures, including laparoscopic, laparoscopically assisted, and robot-assisted approaches, are utilized in major abdominal operations to attenuate the surgical stress response, decrease postoperative complications, and enhance patient satisfaction (2–4). Despite these advances, such procedures still induce substantial surgical trauma, triggering neuroendocrine, inflammatory, and metabolic stress responses, with in-hospital mortality rates of up to 4% and severe complication rates of approximately 15% (5). Consequently, patients often experience impaired physiological recovery and a high risk of complications, eventually contributing to a significant long-term reduction in quality of life (6, 7). Therefore, timely interventions in this patient population are essential to improve quality of postoperative recovery. The Quality of Recovery-15 (QoR-15) is a validated patient-reported measure assessing psychological support, physical comfort, independence, emotional state, and pain (8, 9). Lower scores are associated with worse postoperative outcomes. Key determinants consist of anesthesia type, surgical factors, patient features, and complications, with postoperative pain, nausea, vomiting, and opioid use being major contributors (8).

Current strategies rely on multimodal, multidisciplinary approaches, including optimized perioperative care and advanced anesthetic techniques such as minimally invasive operation, goal-directed fluid treatment, multimodal analgesia, and regional anesthesia, aiming to reduce stress response and complications while enhancing recovery (10, 11). However, despite these advances, a substantial proportion of patients still experience suboptimal postoperative recovery. Repetitive transcranial magnetic stimulation (rTMS) is a noninvasive neuromodulatory method that utilizes magnetic fields produced by electrical currents to activate the cerebral cortex (12, 13). RTMS can alter cortical excitability and network dynamics; however, the direction and magnitude of these effects are not determined by stimulation frequency alone. Although low- and high-frequency protocols have traditionally been associated with inhibitory- and facilitatory-like effects, respectively, physiological responses may also vary according to stimulation intensity, pulse number, stimulation pattern, target localization, treatment schedule, and individual neurophysiological state (14, 15). Accumulating evidence suggests that rTMS can modulate dysfunctional neural circuits and promote neuroplasticity (16). The dorsolateral prefrontal cortex (DLPFC) is critically involved in cognitive and affective processing, including attention, memory, emotional regulation, and pain perception, making it a commonly targeted region in rTMS protocols (17–22). Stimulation of the DLPFC should therefore not be understood as acting through an isolated cortical region or a series of separate linear pathways. Instead, DLPFC-targeted rTMS may influence remote cortical and subcortical regions through connectivity-dependent and top-down modulation of distributed networks, including prefrontal–cingulate and prefrontal–limbic systems. Network-based accounts of prefrontal neuromodulation further suggest that stimulation may influence functions beyond the directly targeted cortical region by modifying attentional control, sensory integration, and interactions between cognitive and affective systems (23). The direction, magnitude, and duration of these effects may vary according to stimulation frequency, intensity, pulse number, session number, treatment timing, target localization, individual anatomy, and baseline network state. Accordingly, the potential effects of preoperative DLPFC-rTMS on pain, emotional distress, physical comfort, and functional recovery may reflect interrelated effects of distributed network modulation rather than separate or established causal pathways. These network-level considerations provide a plausible rationale for evaluating a multidimensional patient-reported outcome such as the QoR-15.

Multidomain clinical changes have been reported following repeated-session DLPFC-rTMS protocols, including improvements across cognitive, affective, and functional domains in prolonged stimulation paradigms (24). However, such findings should not be extrapolated directly to the present single-session perioperative protocol. Instead, the intervention is hypothesized to induce transient modulation or priming of distributed networks that may persist beyond the stimulation period and remain relevant during early postoperative recovery (25, 26). Whether these short-term changes persist through anesthesia and surgical stress and are sufficient to influence the QoR-15 score remains uncertain and constitutes an exploratory mechanistic premise of this trial. Because postoperative recovery is multidimensional, modest concurrent changes in pain, emotional well-being, physical comfort, and functional recovery may collectively contribute to an improvement in patient-perceived recovery. The QoR-15 integrates these interrelated physical and emotional dimensions into a single patient-centered measure and may therefore capture such multidomain changes (27). In addition, rTMS has been associated with molecular changes related to neuroplasticity, including BDNF (28), whereas circulating NfL is increasingly recognized as a marker of neuroaxonal injury and has been associated with postoperative delirium and perioperative neuronal injury (29). Accordingly, BDNF and NfL will therefore be evaluated as exploratory biological correlates rather than established mediators of the clinical effect.

Despite its therapeutic potential, high-quality randomized controlled trials estimating the rTMS effect on quality of postoperative recovery—particularly as measured by patient-centered outcomes such as the QoR-15—are lacking. Few randomized controlled trials have specifically examined the rTMS effects on quality of postoperative recovery using validated patient-reported outcome measures (e.g., the QoR-15). Therefore, this investigation seeks to address this gap by evaluating clinical efficacy; exploratory analyses will assess potential biological correlates. We hypothesize that adjunctive rTMS, compared with sham stimulation, will result in improved postoperative recovery outcomes in patients undergoing major abdominal surgery. Sham stimulation is selected as the comparator to control for placebo effects and ensure blinding integrity. The primary objective is to determine whether preoperative rTMS improves the QoR-15 score at 24 h after surgery. All secondary clinical outcomes will be considered exploratory and hypothesis-generating, while BDNF and NfL will be evaluated as exploratory biological correlates. The conceptual framework and overall study timeline, including the hypothesized neuromodulatory pathways, clinical outcomes, and exploratory biomarker assessments, are illustrated in Figures 1, 2.

**Figure 1:**
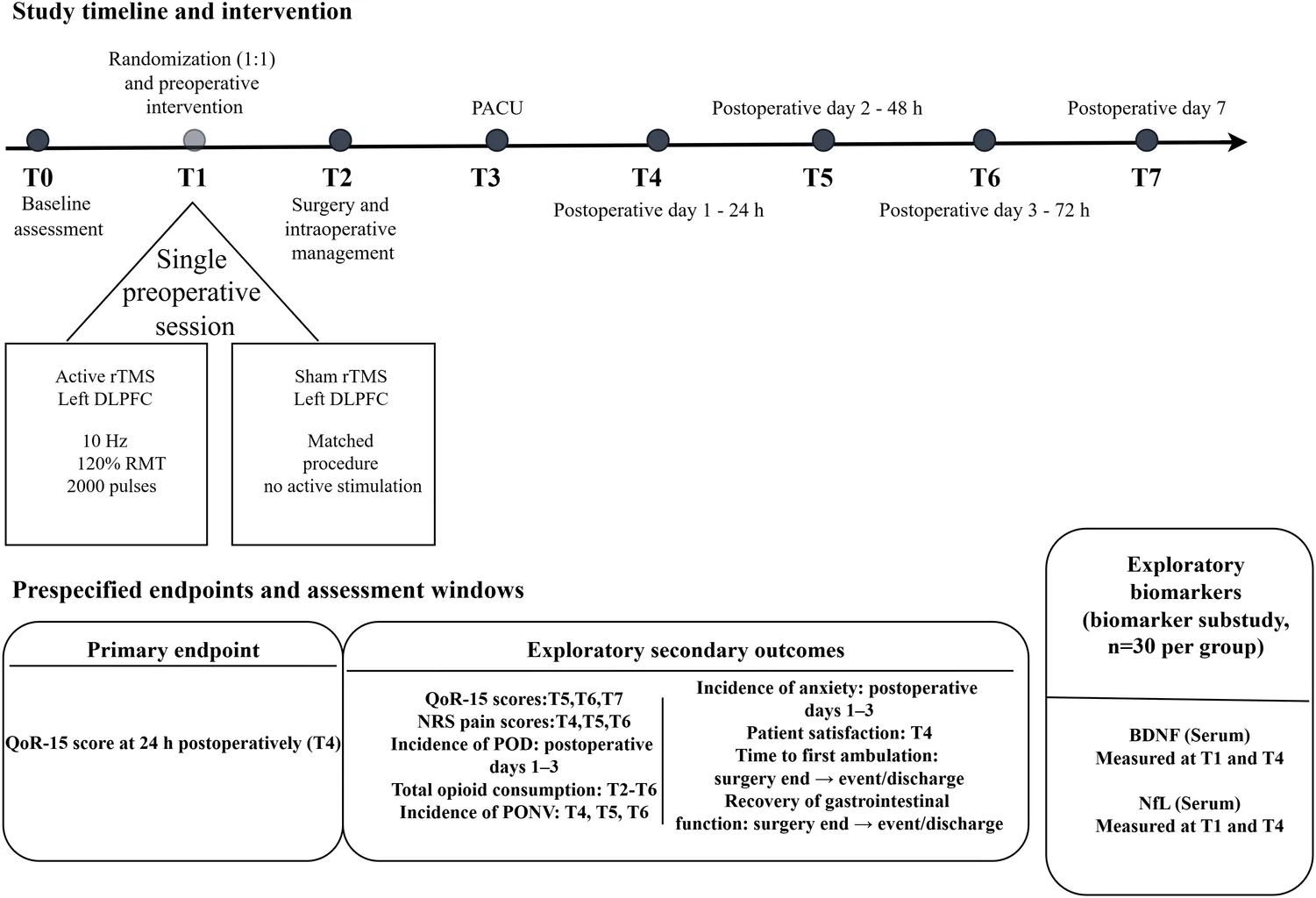
Study timeline and outcome assessment schedule. PACU, post-anesthesia care unit; POD, postoperative delirium; PONV, postoperative nausea and vomiting; QoR-15, Quality of Recovery-15; NRS, numeric rating scale; rTMS, repetitive transcranial magnetic stimulation; RMT, resting motor threshold; DLPFC, dorsolateral prefrontal cortex; NfL, neurofilament light chain.

**Figure 2:**
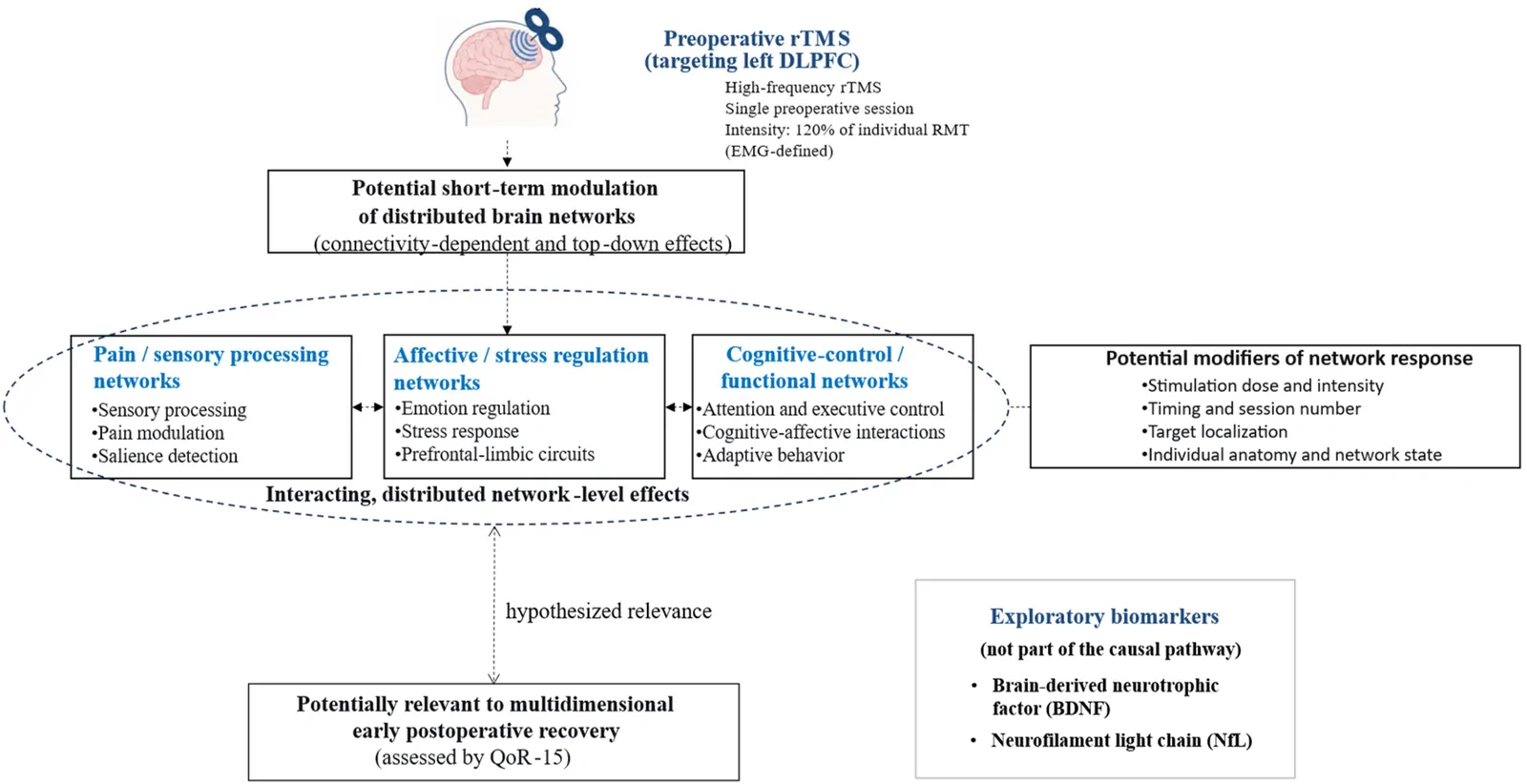
Conceptual framework for the hypothesized network-level effects of preoperative rTMS. A single preoperative session of high-frequency rTMS targeting the left DLPFC is hypothesized to transiently modulate interacting distributed cortical and subcortical networks involved in pain and sensory processing, affective and stress regulation, and cognitive-control and functional processes. Dashed connectors indicate hypothesized relevance or interaction and do not represent established causal or mediating pathways. The magnitude and duration of these potential effects may vary according to stimulation dose and intensity, timing and session number, target localization, and individual anatomy and network state. These network-level processes are hypothesized to be relevant to multidimensional early postoperative recovery assessed by the QoR-15. BDNF and NfL are shown separately as exploratory biological correlates and are not tested as mediators of the clinical effect.

## 2 Methods and analysis

### 2.1 Study design

This study is a prospective, single-center, randomized controlled trial carried out at Jiaxing University Affiliated Hospital. A total of 208 participants listed for major abdominal surgery will be enrolled. The Ethics Committee of Jiaxing University Affiliated Hospital, on January 6, 2026 (2026-LP-003), has given its approval to the protocol that has been registered on February 25, 2026, with the China Clinical Trial Registry (ChiCTR2600119250). We will enroll participants according to the inclusion and exclusion criteria. The investigation will be conducted in accordance with the CONSORT statement, and informed consent will be provided by all participants before enrollment. The protocol has been developed following SPIRIT 2013 guidelines, and Figure 3 displays the study flow.

**Figure 3:**
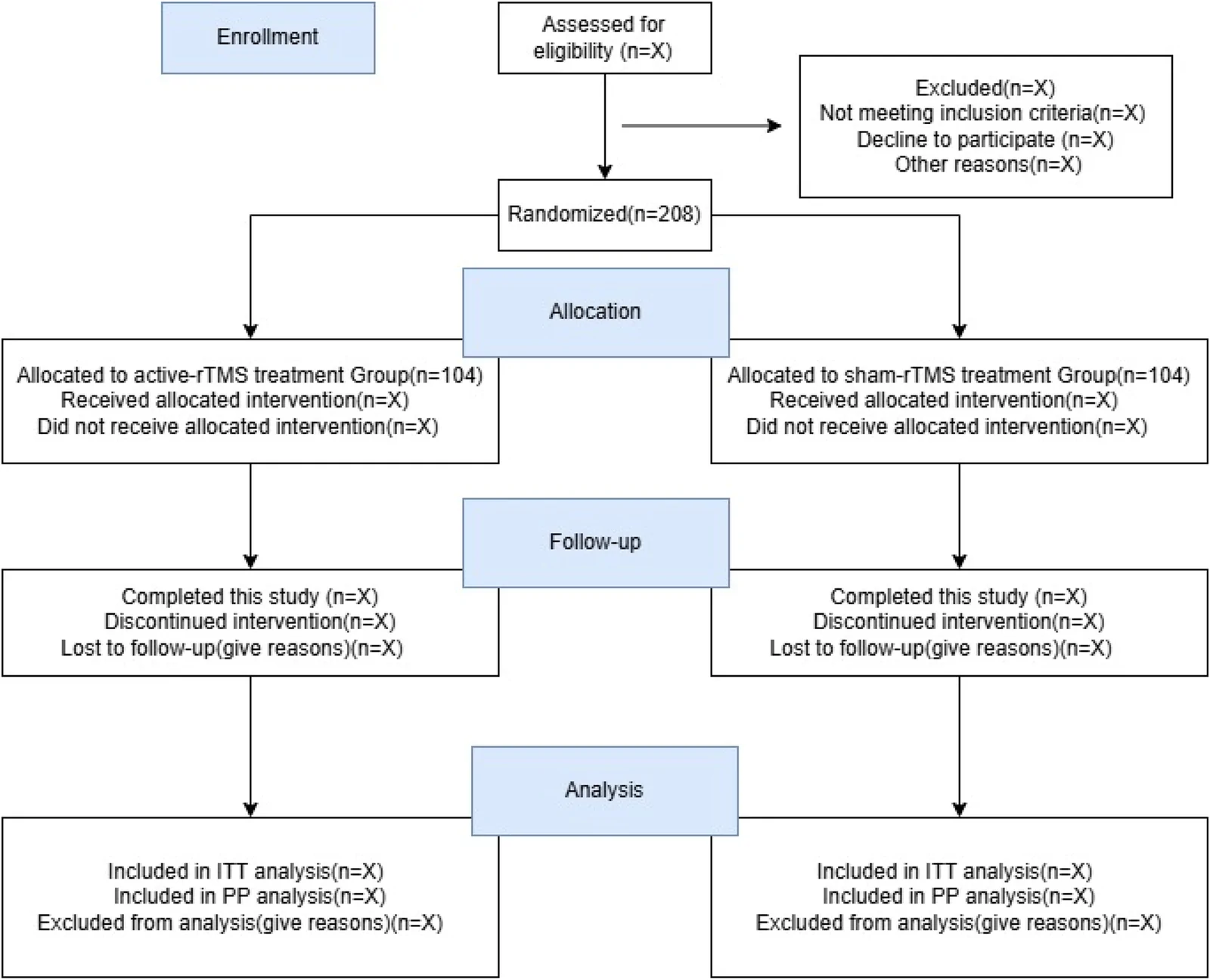
The study's flow diagram. PP, per-protocol, ITT, intention-to-treat, rTMS, Repetitive transcranial magnetic stimulation.

### 2.2 Eligibility criteria

#### 2.2.1 Inclusion and exclusion criteria

**Inclusion criteria:**

1. Scheduled to undergo laparoscopic major abdominal surgery (e.g., radical colorectal cancer resection or subtotal gastrectomy).
2. American Society of Anesthesiologists (ASA) physical status II–III.
3. Age ≥18 years.
4. Ability to understand study procedures and communicate with research staff.
5. Written informed consent provided.

**Exclusion criteria:**

1. Intracranial metallic implants or cardiac pacemakers.
2. History of epilepsy or other significant neurological disorders.
3. Current or past major psychiatric disorders or use of psychotropic medications.
4. Preoperative cognitive impairment (MMSE below education-adjusted cutoffs: ≤17 illiterate, ≤20 primary school, ≤22 middle school, ≤23 high school/college).
5. History of significant head or brain injury.
6. Severe cardiovascular or cerebrovascular disease (e.g., unstable coronary artery disease, recent myocardial infarction, NYHA III–IV heart failure, uncontrolled hypertension >180/110 mmHg, or recent stroke/TIA).
7. Substance abuse or dependence (including alcohol or psychoactive medications).
8. Chronic pain poorly controlled for ≥1 month.
9. Known allergy to anesthetic agents or study medications.
10. Prior exposure to transcranial magnetic stimulation within three months.

### 2.3 Withdrawal or dropout criteria

Based on the principles outlined in the Declaration of Helsinki, participants may withdraw from the trial at any time without providing a reason. We will ensure continuous care, and withdrawal reasons will be recorded.

Investigators may discontinue participation if any of the following occurs:

1. Ethical or medical concerns requiring termination of participation;
2. Major protocol deviations or non-adherence;
3. Occurrence of a serious adverse event (AE);
4. Use of concomitant medications that could interfere with outcomes;
5. Loss to follow-up (defined as inability to complete QoR-15 assessment after repeated contact attempts within 7 days postoperatively, including telephone re-contact within 2 additional days);
6. Participant withdrawal of consent or refusal of the study intervention, with reasons recorded on a “Withdrawal/Change of Status” form.

### 2.4 Randomization and blinding

Participants will be randomly assigned in a 1:1 ratio to active or sham rTMS using a computer-generated, stratified permuted-block randomization sequence. Randomization will be stratified by predefined surgical procedure type (colorectal surgery vs. gastric surgery) to reduce potential imbalance in surgical stress between treatment groups. Within each surgical stratum, randomly varying block sizes of 4 and 6 will be used to maintain allocation concealment and approximate balance between treatment groups. The allocation sequence will be generated and maintained by an independent researcher who will not participate in recruitment, intervention delivery, outcome assessment, data entry, or statistical analysis. Separate sequentially numbered, sealed, opaque envelopes will be prepared for each surgical stratum and stored securely by designated personnel. After eligibility and surgical stratum have been confirmed, the next sequential envelope within the corresponding stratum will be opened immediately before the intervention.

Due to the nature of the intervention, the rTMS interventionist cannot be blinded to treatment allocation, whereas participants and outcome assessors will remain blinded throughout outcome assessment. To maintain blinding, the active and sham interventions will be matched as closely as possible in appearance and procedural delivery. Outcome assessments will be conducted by independent assessors who will receive standardized training in blinding procedures and outcome assessment criteria before study initiation. To minimize potential performance and assessment bias arising from the interventionist's knowledge of treatment allocation, the interventionist will not participate in perioperative clinical decision-making, postoperative outcome assessment, questionnaire administration, data entry, or statistical analysis. Furthermore, participant–interventionist interactions will follow a standardized procedural script and workflow in both groups to minimize differential behavioral cues.

To assess the effectiveness of blinding and the potential for bias arising from treatment awareness, blinding success will be formally evaluated in both participants and outcome assessors. Immediately after completion of the primary QoR-15 assessment at 24 h postoperatively (T4) and before formal disclosure of treatment allocation, participants and the corresponding outcome assessors will independently indicate whether they believe the participant received active rTMS, sham rTMS, or are unsure. They will also rate their confidence in the guess using a 5-point Likert scale ranging from 1 (not confident at all) to 5 (extremely confident). Responses will be collected independently by research personnel who are not involved in intervention delivery or outcome assessment. Figure 4 presents the specific details of this evaluation.

**Figure 4:**
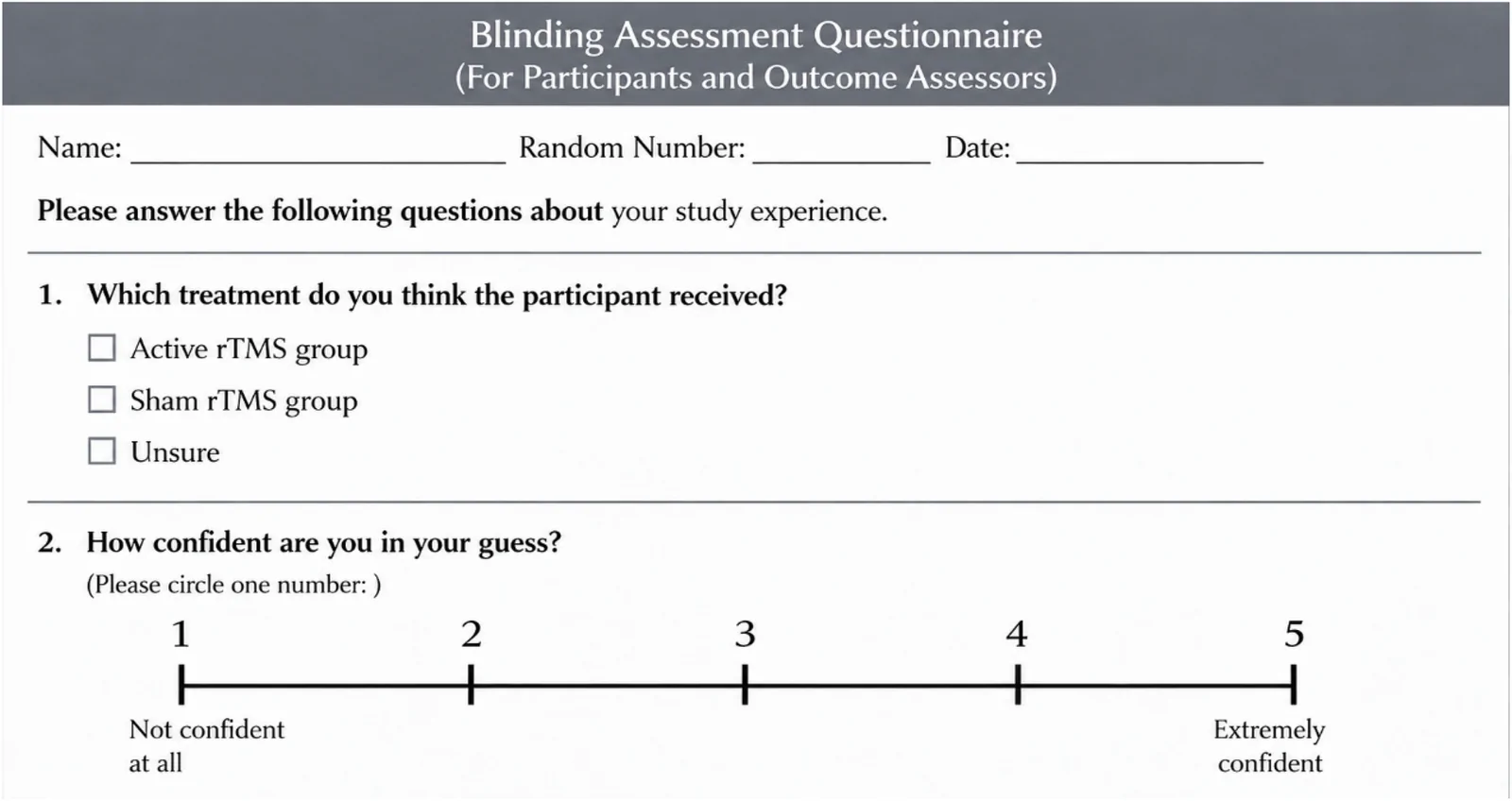
Blinding assessment questionnaire for participants and outcome assessors: the questionnaire will be completed independently by the participant and the corresponding blinded outcome assessor after completion of the 24-h primary outcome assessment and before formal disclosure of treatment allocation.

### 2.5 Unblinding

During the intervention phase, participants will be systematically monitored for potential complications and serious AEs (SAEs) associated with rTMS. Potential AEs include tinnitus, dizziness, headache, nausea, arrhythmia, vomiting, or seizures, with emergency unblinding permitted only if critical for clinically managing a SAE.

### 2.6 Anesthesia

Upon arriving in the operating room, we will establish intravenous access, central venous catheterization and initiate standard monitoring [e.g., heart rate, electrocardiography (ECG), invasive arterial blood pressure, pulse oximetry (SpO_2_), and bispectral index (BIS)]. After adequate collateral circulation has been confirmed using the Allen test, radial artery cannulation will be performed under local lidocaine anesthesia for continuous blood pressure monitoring and arterial blood gas analysis. Central venous catheterization will be performed for fluid and medication administration.

General anesthesia will be induced with propofol (1.5–2.0 mg/kg), sufentanil (0.1–0.5 μg/kg), and vecuronium (0.1–0.2 mg/kg). Following endotracheal intubation, anesthesia will be maintained using remifentanil (8–10 μg/kg/h) and sevoflurane, with the bispectral index (BIS) maintained between 40 and 60. Hemodynamic management, including fluid administration and vasoactive support, will be performed according to intraoperative clinical requirements and the judgment of the attending anesthesiologist.

To improve postoperative analgesia and minimize postoperative nausea and vomiting (PONV), hydromorphone hydrochloride (0.5 mg) and ondansetron hydrochloride (4 mg) will be administered intravenously approximately 30 min before the completion of surgery. To reduce potential confounding effects on postoperative recovery outcomes, other perioperative regional anesthesia techniques, including epidural analgesia and erector spinae plane block, will not be permitted except for transversus abdominis plane block. Likewise, routine perioperative administration of nonsteroidal anti-inflammatory drugs (NSAIDs) or acetaminophen will be avoided unless rescue analgesia is required. After surgery, patients will be transferred to the post-anesthesia care unit (PACU) or intensive care unit (ICU), depending on their postoperative clinical status. Postoperative analgesia will be provided via intravenous patient-controlled analgesia (PCA) containing sufentanil (1.5 μg/kg) and ondansetron (4 mg), diluted with normal saline to a final volume of 100 mL. The PCA device will be set with a continuous infusion rate of 2 mL/h, a bolus dose of 0.5 mL, a lockout interval of 15 min, and a maximum infusion limit of 10 mL/h.

Postoperative pain intensity will be evaluated using the numerical rating scale (NRS, 0–10). Rescue analgesia will be administered when the NRS score is ≥4 at rest or during movement. Intravenous flurbiprofen axetil (50 mg) or acetaminophen will be used as first-line rescue analgesics according to institutional protocols. Additional opioid analgesics may be administered at the discretion of the attending physician in cases of persistent moderate-to-severe pain despite rescue treatment. Routine PONV prophylaxis will include intraoperative ondansetron administration. Patients experiencing postoperative nausea or vomiting will receive rescue antiemetic treatment with intravenous metoclopramide (10 mg), as clinically indicated. Intraoperative and postoperative analgesic regimens will be standardized according to a predefined protocol, and any deviations will be documented and adjusted for in the statistical analysis.

### 2.7 Study interventions

rTMS will be prescribed by a credentialed clinician and will be administered under the supervision of a trained healthcare professional. All personnel have been trained in device operation, coil positioning, and motor threshold assessment, and have been certified in AE management, seizure response, and basic life support (30). On the day of surgery, before anesthesia, participants will receive a single session of active or sham stimulation targeting the DLPFC in the pre-anesthesia room (Table 1). We will first measure the patient's resting motor threshold (RMT). Active rTMS will be delivered at 120% of the resting motor threshold (RMT) and 10 Hz for approximately 20 min (31, 32). The procedure will be administered and monitored by a trained investigator, and stimulation will be discontinued immediately in the event of intolerable discomfort.

**Table 1:** List of enrollment, outcome assessments, interventions, and data collection.

Assessment	Study period							
	T0	T1	T2	T3	T4	T5	T6	T7
Enrollment and allocation								
Eligibility screening	X							
Informed consent	X							
Demographic data	X							
Baseline characteristics	X							
Randomization		X						
Allocation		X						
Intervention								
Active rTMS		X						
Sham rTMS		X						
Primary outcome								
QoR-15 score at 24 h					X			
Exploratory secondary outcomes								
QoR-15 scores						X	X	X
NRS pain scores					X	X	X	
Postoperative delirium					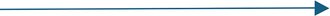			
Cumulative opioid consumption			X	X	X	X	X	
PONV					X	X	X	
Postoperative anxiety					X	X	X	
Patient satisfaction					X			
Time to first ambulation					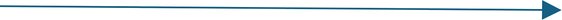			
Time to first flatus				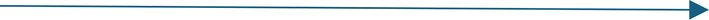				
Exploratory outcomes								
Serum BDNF		X			X			
Serum NfL		X			X			
Safety assessments								
Adverse events	Throughout the study period until discharge							

In accordance with the SPIRIT 2013 Statement for typical protocol items. T0:1 day of pre-surgery T1:1 h of pre-surgery T2: During surgery T3: During PACU T4:24 h post-operation T5: 48 h post-operation T6:72 h post-operation T7: 7d post-operation; PONV, postoperative nausea and vomiting; POD, Postoperative delirium; PACU, post-anesthesia care unit; QoR-15, Quality of Recovery-15; NRS, Numerical Rating Scale; BDNF, brain-derived neurotrophic factor; AEs, adverse events; NfL, neurofilament light chain.

#### 2.7.1 Intervention methods

The rTMS device used in this study will be the CCY-1 model. Stimulation parameters and target location will be determined before treatment. Resting motor threshold will be determined using single-pulse TMS over the left primary motor cortex, with surface electromyography recorded from the right abductor pollicis brevis muscle while the participant remains fully relaxed. After identification of the motor hotspot corresponding to the right abductor pollicis brevis, RMT will be defined as the lowest stimulator intensity capable of eliciting motor-evoked potentials with a peak-to-peak amplitude of at least 50 μV in at least 5 of 10 consecutive trials. Background electromyographic activity will be monitored throughout threshold determination to confirm adequate muscle relaxation (33). Motor-threshold determination will be performed by trained personnel using standardized participant positioning, electrode placement, coil positioning, and thresholding procedures. The individual RMT and corresponding stimulator output will be documented before treatment. Active rTMS will subsequently be delivered at 120% of the individual EMG-defined RMT. Based on the international 10–20 electroencephalography electrode placement system, the left DLPFC will be localized using the Beam F3 method, an anatomical landmark-based scalp measurement approach that provides an individualized estimation of the F3 electrode site while accounting for individual head circumference. Specifically, the F3 position will be determined using measurements from the vertex (Cz) and the mid-frontal point (Fz) along the nasion–inion midline, followed by a lateral displacement corresponding to 20% of the arc distance from Fz to the left preauricular point. This MRI-free targeting approach has been widely adopted for left DLPFC localization in rTMS protocols (34). Participants will wear earplugs to minimize acoustic discomfort during stimulation. The procedure will be strictly monitored, and stimulation will be terminated immediately in the event of intolerable discomfort, including headache, tinnitus, significant anxiety, or distress. To avoid potential modulation of cortical excitability, no sedative medications, including benzodiazepines, will be administered before stimulation. All rTMS procedures will be conducted in accordance with established international safety guidelines.

#### 2.7.2 Active-rTMS

A figure-of-eight coil will be utilized, with the left DLPFC serving as the stimulation target. The stimulation coil will be localized tangentially over the target scalp region, with the handle oriented posteriorly at approximately 45° to the midline, in accordance with established rTMS protocols. rTMS will be delivered at 120% of the RMT and 10 Hz. Each session will comprise 50 pulses/train (25 s interval) for 40 trains (i.e., 2,000 pulses/session) (35, 36).

#### 2.7.3 Sham rTMS

A sham coil will be used with an identical stimulation site and parameters. The sham stimulation will not deliver active magnetic stimulation but will produce auditory and somatosensory effects (noise and vibration) similar to those generated during active stimulation.

#### 2.7.4 Relevant concomitant care and interventions

To ensure safety and outcome validity, concomitant treatments will be strictly regulated. Medications that significantly lower the seizure threshold (e.g., clozapine, high-dose bupropion) will be prohibited. Alcohol consumption will be prohibited for 24 h before and after each session. Illicit substance use will be assessed and excluded through screening. All medication use will be documented prospectively, including name, dosage, route, frequency, and indication.

#### 2.7.5 Provisions for post-trial care

After operation, we will transfer patients to the PACU or ICU and then to the general ward. Each participant will receive standard postoperative care from a multidisciplinary team, regardless of group allocation. In case of intervention-related harm or serious adverse events (e.g., rTMS-associated injury), necessary medical treatment will be provided free of cost in accordance with insurance policies and applicable laws. Postoperative follow-up visits will be conducted to assess safety, identify complications, and ensure appropriate clinical management until discharge.

### 2.8 Outcomes

#### 2.8.1 Primary outcome

The QoR-15 score at 24 h postoperatively is the primary outcome. This method will assess postoperative recovery across five fields: emotional state, physical comfort, independence, psychological support, and pain (15 items) (8). The range of total scores is 0–150, with better recovery quality indicated by higher scores (37).

#### 2.8.2 Exploratory secondary outcomes

All secondary outcomes will be considered exploratory and hypothesis-generating and will not be interpreted as independently confirmatory efficacy endpoints.

1. QoR-15 scores at T5, T6, and T7: Evaluations will be conducted at three predefined timepoints: 48 h (T5), 72 h (T6), and postoperative day 7 (T7). All evaluations will be conducted by trained personnel blinded to group allocation.
2. Pain scores: We will estimate pain intensity via an 11-point Numeric Rating Scale (NRS; 0 = no pain, 10 = worst pain) at 24 h (T4), 48 h (T5), and 72 h (T6) postoperatively. Pain will be estimated separately at rest and during standardized movement, including coughing, turning in bed, and early ambulation. The highest pain score during movement will be recorded.
3. The incidence of postoperative delirium (POD) will be assessed within the initial three postoperative days (postoperative days 1–3) or until hospital discharge, whichever happens first: Assessments will be performed two times daily at predefined timepoints (08:00–10:00 and 16:00–18:00) via trained personnel blinded to group allocation. Consciousness level will be first evaluated via the Richmond Agitation–Sedation Scale (RASS) (38); participants with a RASS score ≥−3 will undergo delirium assessment via the 3-Minute Diagnostic Interview for CAM (3D-CAM) in general wards or the Confusion Assessment Method for the Intensive Care Unit (CAM-ICU) in ICU settings (39, 40).
4. Cumulative opioid intake: Represented as intravenous morphine milligram equivalents (MME), will be calculated at T2, T3, 24, 48, and 72 h. All opioid doses administered via PCA or as rescue analgesia will be recorded from electronic medical records. Conversion to MME will be performed using standardized equianalgesic conversion tables. Both scheduled and as-needed opioid administrations will be included in the cumulative total (41).
5. Prevalence and severity of postoperative nausea and vomiting (PONV): Evaluated via a Likert scale (none, mild, moderate, or severe) at 24, 48, and 72 h postoperatively.
6. Postoperative anxiety will be assessed: Perioperative anxiety will be assessed at 24, 48, and 72 h postoperatively via the Hospital Anxiety and Depression Scale–Anxiety (HADS-A). The HADS-A is a validated seven-item instrument for assessing psychological distress, with established sensitivity and specificity. A score ≥ 8 will indicate clinically significant anxiety.
7. Patient satisfaction: Patient satisfaction with overall perioperative care will be assessed at 24 h postoperatively (T4) using a single-item 11-point numerical rating scale ranging from 0 (“completely dissatisfied”) to 10 (“completely satisfied”), with higher scores indicating greater satisfaction. The assessment will be conducted by a trained outcome assessor blinded to treatment allocation.
8. Serum biomarkers (e.g., BDNF and NfL) will be assessed in a subset of participants (*n* = 30 per group) as exploratory outcomes. Venous blood (serum) samples will be collected from 30 randomly selected participants per group at T1 before the intervention and at T4, 24 h after surgery. Samples will be promptly processed and kept at −80 °C. Serum levels of BDNF and NfL will be measured using validated immunoassays (e.g., ELISA for BDNF and ultra-sensitive single-molecule array for NfL).
9. Time to initial ambulation: The time to initial ambulation will be defined as the interval from the end of the operation to the first successful out-of-bed mobilization, operationalized as standing and walking a minimum distance of 5 m, with or without assistance. Time to ambulation will be recorded in hours by trained nursing staff using standardized case report forms (CRFs). Assisted ambulation will be allowed and documented (42).
10. Gastrointestinal recovery will be assessed using time to first flatus. This will be defined as the interval (in hours) from the end of operation to the first passage of flatus, as reported by the patient and confirmed by nursing staff, with assessments performed every 8 h.

### 2.9 AEs and safety

AEs will be defined as any untoward medical occurrences in study participants. Potential rTMS-related AEs, including headache, nausea, dizziness, vomiting, tinnitus, arrhythmia, and seizures, will be recorded, controlled, and followed until resolution or stabilization, with causality assessed. AEs and SAEs will be coded, summarized, and documented in subsequent publications. SAEs and unexpected AEs will be rapidly documented to the ethics committee and relevant regulatory authorities, which will retain the authority to recommend trial suspension or termination. Given the established safety experience with rTMS when administered according to recommended protocols, the anticipated intervention-related risk is considered low. Participant safety and data integrity will be further ensured through independent data follow-up. An independent Data Monitoring Committee (DMC) will supervise trial conduct and periodically review accumulating safety and efficacy data during the study. The committee will comprise three independent experts, including an anesthesiologist, a biostatistician, and a clinical research specialist, none of whom will participate in patient recruitment, intervention delivery, outcome assessment, or statistical analysis. The responsibilities and operational procedures of the DMC will be predefined in a formal charter, including meeting schedules, safety monitoring procedures, and recommendations regarding trial continuation or termination. All serious adverse events (SAEs) will be recorded throughout the study and reported to the principal investigator and institutional ethics committee within 24 h of recognition. Additional safety reviews may be convened at the request of the DMC when clinically necessary. Trial discontinuation will be considered if unexpected intervention-related SAEs occur disproportionately in the intervention group or if continued participation is judged to pose unacceptable risk to patient safety.

### 2.10 Data collection

All data will be documented in standardized CRFs. Preoperative data will include demographic characteristics, medical history, baseline health status, planned surgical procedure, educational level, socioeconomic status, family history of malignancy, body mass index (BMI), and ASA classification. Intraoperative data will include blood loss, urine output, fluid administration, and durations of operation and anesthesia. Postoperative data will include the postoperative anxiety (assessed using the HADS-A), pain intensity (evaluated via the numerical rating scale, NRS), occurrence of PONV and patient satisfaction (assessed using a single-item 11-point numerical rating scale). At study completion, data will be assessed by an independent, trained statistician blinded to group allocation. Personnel accounting for perioperative follow-up and the collection of blood samples will not participate in the intervention and will be blinded to group allocation. After collection, samples will be centrifuged, processed, and transported to a certified laboratory for analysis. An independent investigator will collect questionnaire data at all time points, initially recording them on paper CRFs before transcription into electronic databases. In accordance with regulatory necessities, biological samples will be kept in a −80 °C freezer with restricted access. Data accessibility will be limited to authorized personnel only.

### 2.11 Sample size

We will assess the sample size depending on the minimal clinically important difference (MCID) of the QoR-15 score, with parameters informed by preliminary pilot data. Assumptions regarding variability will be derived from both pilot observations and previously published studies. Depending on findings from the pilot investigation, the mean QoR-15 score at 24 h postoperatively will be anticipated to be 108.27 [standard deviation (SD) = 14.92] in the active rTMS group and 101.23 (SD = 15.24) in the sham rTMS group. According to the updated MCID for the QoR-15 scale, we will consider a difference of six points as clinically meaningful (43). To determine this variation with a two-sided significance level of *α* = 0.05 and a statistical power of 80%, an estimated sample size of 74 participants/group is necessary. After representing an anticipated dropout rate of 10% and pre-specified subgroup analyses, the sample size will be elevated to 104 participants/group (208 in total), as calculated using PASS15 software. Prespecified subgroup analyses will be considered exploratory and will not be powered for formal interaction testing.

### 2.12 Statistical analysis

The primary analysis will be according to the principle of intention-to-treat (ITT), comprising all randomized participants irrespective of protocol adherence. We will evaluate continuous variables' normality via the Shapiro–Wilk test and histograms' visual inspection. Baseline characteristics will be summarized descriptively. Continuous variables will be presented as mean (SD) or median (IQR), and categorical variables as frequencies and percentages. No formal hypothesis testing of baseline characteristics will be performed; clinically relevant imbalances, if present, will be considered in adjusted analyses.

The primary outcome, defined as the QoR-15 score at 24 h postoperatively, will be analyzed in the intention-to-treat (ITT) population using analysis of covariance (ANCOVA). The 24-h postoperative QoR-15 score will be included as the dependent variable, with treatment allocation entered as the primary fixed effect. The model will be adjusted for baseline QoR-15 score and prespecified covariates, including age, body mass index (BMI), frailty index, comorbidity burden, surgical procedure type, and duration of surgery. Surgical procedure type, corresponding to the randomization stratification factor (colorectal vs. gastric surgery), will be included as a fixed-effect covariate in the final ANCOVA model. Treatment effects will be presented as adjusted mean differences with corresponding 95% confidence intervals.

Missing outcome data in the primary intention-to-treat (ITT) analysis will be handled using multiple imputation by chained equations (MICE) under a missing-at-random (MAR) assumption, rather than complete-case analysis. At least 20 imputed datasets will be generated, with the final number determined according to the proportion of incomplete data. The imputation model will include treatment allocation, baseline QoR-15 score, relevant outcome measurements, prespecified prognostic covariates, and auxiliary variables associated with missingness or outcome values, including age, body mass index, frailty index, comorbidity burden, surgical procedure type, duration of surgery, and postoperative complications. Estimates from the imputed datasets will be combined using Rubin's rules.

To assess the robustness of the primary findings to departures from the MAR assumption, pattern-mixture models with delta adjustment will be used as sensitivity analyses under missing-not-at-random (MNAR) assumptions, particularly for potentially informative missingness associated with postoperative complications or other adverse clinical events. Imputed 24-h QoR-15 values for participants with missing primary-outcome data will be shifted toward clinically plausible less favorable values across a prespecified range of delta values. The delta range will be specified in the statistical analysis plan before database lock and treatment-code unblinding.

Participants experiencing postoperative complications, conversion to open surgery, or reoperation will remain in the primary ITT population. The per-protocol population will include participants who receive the allocated intervention without major protocol deviations, including receipt of an incorrect intervention, insufficient stimulation below the predefined minimum dose, major eligibility violations, or use of prohibited concomitant therapies that may affect the primary outcome. Complete-case and per-protocol analyses will be conducted as additional sensitivity analyses. Additional sensitivity analyses excluding participants undergoing conversion to open surgery or reoperation may also be performed but will not replace the primary ITT analysis.

All secondary-outcome analyses will be exploratory and hypothesis-generating. The prespecified ANCOVA of the 24-h postoperative QoR-15 score will remain the primary analysis. As a complementary exploratory analysis, QoR-15 scores measured at 24, 48, and 72 h and on postoperative day 7 will be analyzed using generalized estimating equations (GEE), including treatment group, time point, and their interaction, with adjustment for baseline QoR-15 score and the covariates prespecified for the primary analysis. Model-based contrasts will be used to estimate adjusted between-group differences at each time point with corresponding 95% confidence intervals. Other repeatedly measured continuous outcomes, including pain intensity and HADS-A scores, will be analyzed using GEE with an appropriate distribution and link function. Repeated binary outcomes, including PONV at individual postoperative time points, will be analyzed using logistic GEE, whereas participant-level binary outcomes, including the occurrence of postoperative delirium within the first three postoperative days, will be analyzed using multivariable logistic regression. Patient satisfaction at 24 h will be analyzed using linear regression. If model assumptions are not adequately met, appropriate transformations, robust methods, or prespecified nonparametric alternatives will be applied. Cumulative postoperative opioid consumption at 24, 48, and 72 h will be analyzed using GEE. Given its potentially right-skewed distribution, a gamma distribution with a log link or an appropriate robust alternative will be used. Time to first ambulation and time to first flatus will be analyzed from the end of surgery using Kaplan–Meier methods and Cox proportional hazards regression, with discharge before the event treated as censoring. Hazard ratios with 95% confidence intervals will be reported, with values greater than 1 indicating earlier recovery. The proportional hazards assumption will be assessed using Schoenfeld residuals; if it is violated, restricted mean survival time analysis will be considered. Effect estimates and 95% confidence intervals will be emphasized for all exploratory secondary outcomes. To address multiplicity, the Benjamini–Hochberg procedure will be applied to the prespecified family of *P* values arising from the main secondary clinical outcome comparisons to control the false discovery rate. Both nominal and Benjamini–Hochberg-adjusted *P* values will be reported. Secondary findings will be interpreted as exploratory and hypothesis-generating irrespective of statistical significance. Missing secondary-outcome data will be handled under a missing-at-random assumption using methods appropriate to the corresponding statistical model.

Blinding success will be evaluated separately in participants and outcome assessors. Treatment guesses (active rTMS, sham rTMS, or unsure) and confidence scores will be summarized descriptively. Bang's Blinding Index (BBI) and corresponding 95% confidence intervals will be calculated separately by treatment group for participants and assessors. Values close to zero will indicate successful blinding, whereas positive values will indicate a tendency toward correct identification of treatment allocation. The proportion of correct guesses will also be reported as a supplementary measure of blinding success.

Exploratory analyses will examine whether changes in BDNF and NfL are associated with postoperative recovery outcomes. These analyses will be considered hypothesis-generating, and no causal conclusions will be drawn. Associations between changes in biomarkers (e.g., BDNF and NfL) and postoperative recovery outcomes (QoR-15 scores, pain intensity, and opioid consumption) will be evaluated using multivariable linear regression models, adjusting for prespecified covariates. Correlation analyses (Pearson or Spearman, as appropriate) will also be performed to assess relationships between biomarker levels and clinical outcomes.

Prespecified subgroup analyses for the primary outcome are considered exploratory and hypothesis-generating. To reduce the risk of false findings and multiplicity inflation, only a limited set of clinically relevant variables will be examined, including age and frailty status. Subgroup effects will be assessed by including interaction terms between treatment allocation and subgroup variables in the regression models. Given the limited statistical power for interaction testing in this single-center trial, all subgroup findings will be interpreted cautiously, and no firm conclusions about treatment-effect heterogeneity will be drawn.

To assess the robustness of the primary outcome findings across different analysis populations, sensitivity analyses will be conducted in the PP populations. In addition, alternative analytical approaches will be applied where appropriate to evaluate the consistency of results.

We will conduct all statistical analyses using SPSS v27.0 (IBM Corp., Armonk, USA) and R software (version 4.3.0; R Foundation for Statistical Computing). All statistical tests will be two-sided. For the primary outcome, statistical significance will be assessed according to the prespecified two-sided alpha level.

## 3 Discussion

This prospective, randomized, sham-controlled trial with patient and assessor blinding will evaluate whether a single preoperative session of high-frequency rTMS improves early postoperative recovery following major abdominal surgery. The QoR-15 was selected as the primary outcome because it captures multidimensional, patient-centered early recovery rather than an isolated postoperative symptom or complication (44). Accordingly, it may be sensitive to modest concurrent changes across several postoperative domains potentially influenced by preoperative rTMS. The sham-controlled design, blinded outcome assessment, and integration of clinical outcomes with exploratory biomarkers will strengthen the methodological rigor of the trial. If a beneficial effect is observed, the findings will provide preliminary evidence supporting further investigation of preoperative rTMS as an adjunct to multimodal perioperative care.

Previous clinical studies provide preliminary evidence that DLPFC-targeted rTMS may influence individual domains of postoperative recovery, including delirium and pain (45). However, these studies differed from the present trial in patient population, surgical setting, stimulation timing, and outcome measures. The present study extends this line of investigation by evaluating whether a single preoperative session of high-frequency rTMS can influence overall early postoperative recovery as assessed by the QoR-15. If a beneficial effect is observed, it may reflect effects across multiple recovery domains, consistent with the hypothesized preoperative “priming” effect described in the Introduction. BDNF and NfL measurements may provide additional exploratory biological context for the clinical findings, but will not be interpreted as evidence of established mediating mechanisms. Because biomarker analyses will be performed in only a subset of participants without a formal power calculation, negative findings should be interpreted cautiously.

From a network-level perspective, any observed clinical effect should not be interpreted as evidence for a single linear pathway linking DLPFC stimulation to postoperative recovery. DLPFC-targeted rTMS may exert connectivity-dependent, top-down influences across distributed cortical and subcortical networks involved in affective regulation, sensory processing, pain modulation, and functional control (23). Importantly, the magnitude and persistence of such effects are likely to depend on stimulation dose, schedule, target localization, and individual network characteristics. Therefore, multidomain effects reported after repeated DLPFC-rTMS should not be directly extrapolated to the present single-session protocol, for which any neuromodulatory effect is expected to be shorter-lived and remains to be established.

This study has several limitations. First, the relatively limited sample size and single-center design may restrict the generalizability of the findings; therefore, larger multicenter studies will be required to validate the results. Second, the intervention consists of a single preoperative rTMS session, whereas most established therapeutic rTMS protocols involve repeated sessions. Although we hypothesize that a single session administered immediately before surgery may induce a short-term neuromodulatory priming effect that is temporally relevant to early postoperative recovery, the magnitude and duration of such effects remain uncertain. The single-session strategy was also selected for pragmatic reasons, because repeated postoperative stimulation may be difficult to implement in patients experiencing pain, fatigue, nausea, hemodynamic instability, or limited mobility. Nevertheless, the single-session design was selected to test whether a temporally targeted preoperative neuromodulatory intervention can influence early postoperative recovery. Accordingly, the present study should be regarded as an exploratory evaluation of a temporally targeted single-session intervention rather than an assessment of an established or optimal rTMS treatment schedule. Future studies should investigate repeated stimulation, different treatment intervals, and dose–response relationships. Third, the intervention will employ fixed stimulation parameters, and possible dose–response links among various parameter combinations have not been systematically investigated (46). Finally, although key confounders have been controlled for, unmeasured variables—such as subtle differences in surgical procedures or variations in supportive care—may influence the outcomes. Therefore, the outcomes should be interpreted with caution and need verification in larger, well-designed multicenter studies.
